# Parameterized Complexity of Eulerian Deletion Problems

**DOI:** 10.1007/s00453-012-9667-x

**Published:** 2012-06-22

**Authors:** Marek Cygan, Dániel Marx, Marcin Pilipczuk, Michał Pilipczuk, Ildikó Schlotter

**Affiliations:** 1 Institute of Informatics, University of Warsaw, ul. Banacha 2, 02-097, Warsaw, Poland; 2 Institut für Informatik, Humboldt-Universität zu Berlin, Unter den Linden 6, 10099 Berlin, Germany; 3 Department of Informatics, University of Bergen, Postboks 7803, 5020 Bergen, Norway; 4 Department of Computer Science and Information Theory, Budapest University of Technology and Economics, Magyar tudósok körútja 2, 1117 Budapest, Hungary

**Keywords:** Fixed-parameter tractability, Kernelization, Eulerian graph, Deletion distance

## Abstract

We study a family of problems where the goal is to make a graph Eulerian, i.e., connected and with all the vertices having even degrees, by a minimum number of deletions. We completely classify the parameterized complexity of various versions: undirected or directed graphs, vertex or edge deletions, with or without the requirement of connectivity, etc. The collection of results shows an interesting contrast: while the node-deletion variants remain intractable, i.e., W[1]-hard for all the studied cases, edge-deletion problems are either fixed-parameter tractable or polynomial-time solvable. Of particular interest is a randomized FPT algorithm for making an undirected graph Eulerian by deleting the minimum number of edges, based on a novel application of the color coding technique. For versions that remain NP-complete but fixed-parameter tractable we consider also possibilities of polynomial kernelization; unfortunately, we prove that this is not possible unless NP⊆coNP/poly.

## Introduction

An undirected graph is Eulerian if it is connected and every vertex has even degree; a directed graph is Eulerian if it is strongly connected and every vertex is balanced (i.e., the indegree equals the outdegree). The class of Eulerian graphs is a well-studied and classical notion in the graph theory. We investigate several algorithmic problems related to the question of how to make a graph Eulerian. We focus on deletion problems, where either vertices or edges can be deleted from the input graph to make it Eulerian, using as few deletions as possible. What makes these problems interesting is the interplay of two different type of constraints: each vertex locally prescribes the constraint that it has to be even/balanced, while retaining connectivity is a global requirement. For comparison, we also investigate the variant of the problem where we have only the local constraints (i.e., the task is to delete the minimum number of edges or nodes to make every vertex even/balanced). As many of the studied problems turn out to be NP-hard, we apply the framework of parameterized complexity to get a more detailed insight.

The investigation of these problems was initiated by Cai and Yang [9] who presented parameterized results for some cases. We complement their work by answering several open questions raised in [9]. Another motivation for our work comes from an observation of Cechlárová and Schlotter [10]: computing the deficiency for a certain type of housing market is equivalent to finding the minimum number of arcs whose deletion makes every strongly connected component of the graph balanced. While we are not able to determine the parameterized complexity of this problem, our results shed light on the complexity of several related ones.

### Related Work

Subgraph problems have been widely studied in the literature. To name a few examples, Lewis and Yannakakis [21] investigated the complexity of the node-deletion problem for hereditary properties, Alon et al. [2] examined edge-deletion problems for monotone properties, while Natanzon et al. [28] and Burzyn et al. [6] studied the classical complexity of edge modification problems for various graph classes.

Subgraph problems have also been looked at from the parameterized perspective. The most extensively studied variants are the node-deletion problems for hereditary properties: the results by Cai [8], and Khot and Raman [18], yield a complete characterization of the fixed-parameter tractable cases. Apart from hereditary properties, FPT algorithms are known for node-deletion problems where the task is to obtain a regular graph [26], a chordal graph [23], a grid [12], etc. Parameterized hardness results have been obtained in numerous cases as well [22, 24]. Recently, researchers focused on the issue of kernelization, yielding both positive [4, 17, 29] and negative results [20].

There is much less known about directed graphs. Raman and Sikdar [32] investigated the parameterized complexity of hereditary node-deletion problems in digraphs, while Raman and Saurabh [31] examined feedback set problems in tournaments. The FPT algorithm by Chen et al. for finding a feedback vertex set in a directed graph [11] resolved a long-standing open question.

Work related to the class of Eulerian graphs mainly concentrated on the extension problem, where the task is to add a minimum number of edges or arcs in order to make the given graph Eulerian. FPT algorithms were given for various settings by Dorn et al. [13] and by Sorge [33]. Eulerian deletion problems were studied by Cai and Yang [9].

### Our Contribution

To settle the classical complexity of the examined problems, first we observe (Theorems 1 and 2) that classical results imply polynomial-time algorithms for the edge-deletion problems where the task is to make the given graph even/balanced: in the undirected case, this is essentially a *T*-join problem, while the directed case can be reduced to a flow problem. These observations answer a question raised by Cai and Yang [9], who observed that the analogous node-deletion problems are NP-hard. Moreover, the aforementioned algorithms are used as subroutines in our FPT results.

By contrast to the polynomial time algorithms, we show that the seemingly similar edge- (or arc-) deletion problems where we aim for an Eulerian graph are NP-hard, even in the extremely restricted case when the input is a cubic planar graph and the number of deletions can be arbitrary (Theorem 3). We investigate both the undirected and the directed cases of Eulerian edge-deletion problem thoroughly from the parameterized point of view: we present a fixed-parameter tractable algorithm for both cases where the parameter is the number of deletions allowed (Theorem 4), and prove that these problems do not admit a polynomial-size kernel unless NP⊆coNP/poly (Theorem 5), which is known to imply a collapse of the polynomial hierarchy to its third level [7, 34]. The FPT results use a novel argument that might be of independent interest. Intuitively, we need to find a solution *S* to a *T*-join problem and a witness (disjoint from *S*) certifying that the graph remains connected after the removal of *S*. Using a random coloring, we partition the edges into two types: each edge can contribute either to the solution or to the witness of the solution. This partition ensures that the solution and the witness are disjoint. While the use of random colorings is a standard technique for finding a solution consisting of disjoint objects [3], we use this technique to separate the solution from its proof of feasibility.

The undirected node-deletion problems, where the task is to obtain an Eulerian or an even graph, were already handled by Cai and Yang [9] who proved their W[1]-hardness. We complemented these results by showing W[1]-hardness for the directed cases as well in Theorem 7. Additionally, we also focus on a slight modification of the node-deletion problems where certain forbidden vertices are not allowed to be deleted. Theorem 8 shows that each of the four node-deletion problems remains W[1]-hard, even if we are only allowed to delete vertices of degree at most 4. This contrasts the easy FPT algorithm applicable if the parameter is not only the number of deletions but also the maximum degree of the graph (Theorem 11).

Table 1 shows a summary of our main results.

**Table 1:** Summary of the main results. Parameterized results only appear when the corresponding problem is NP-hard; the parameter considered is the number of deletions allowed

	Undirected even	Undirected Eulerian	Directed balanced	Directed Eulerian
Vertex deletion:	W[1]-hard [9]	W[1]-hard [9]	W[1]-hard Theorem 7	W[1]-hard Theorem 7
Edge deletion:	P Theorem 1	FPT, no poly kernel Theorems 3, 4, 5	P Theorem 2	FPT, no poly kernel Theorems 3, 4, 5

### Organization of the Paper

Section 2 describes our notation, and provides basic concepts of parameterized complexity. Section 3 discusses polynomial-time solvable edge-deletion problems. We deal with the NP-hard Eulerian edge-deletion problems in Sect. 4, first covering the issue of NP-completeness, and then fixed-parameter tractability and kernelization. Node-deletion problems are discussed in Sect. 5. We summarize our results and draw conclusions in Sect. 6.

## Notation and Preliminaries

Given a graph *G*, let *V*(*G*) denote its vertex set and *E*(*G*) denote its edge set (or, in the directed case, its arc set). The *degree* of a vertex *v* in an undirected graph *G* is denoted by *d*_*G*_(*v*); we say that *v* is *even*, if *d*_*G*_(*v*) is even. For a vertex *v* in a directed graph *G*, we denote by $d_{G}^{in}(v)$ and $d_{G}^{out}(v)$ its indegree and its outdegree, respectively. We say that *v* is *balanced*, if $d_{G}^{in}(v) = d_{G}^{out}(v)$. We define the *degree* of *v* in *G* (where *G* is directed), as $d_{G}(v) = d_{G}^{in}(v) + d_{G}^{out}(v)$; whenever we discuss the maximum degree of a directed graph, we refer to this notion. If *G* is clear from the context, we might omit the subscript. A directed graph is *weakly connected* if the underlying undirected graph is connected. A directed graph is *strongly connected* if for every two vertices *v*,*w* there is a path from *v* to *w*. An *even (balanced) graph* is an undirected (directed) graph where each vertex is even (balanced). An undirected Eulerian graph is a connected even graph, and a directed Eulerian graph is a strongly connected balanced graph.^1^ A straightforward degree counting argument shows that a balanced directed graph is weakly connected if and only if it is strongly connected.

Given a path *P* in a (directed or undirected) graph, the *internal vertices* of *P* are the vertices lying on *P* except for the two end-vertices. If *d*_*G*_(*v*)=2 holds (meaning $d^{in}_{G}(v) = d^{out}_{G}(v) =1$ in the directed case) for each internal vertex *v* of *P*, then we say that the path *P* is an *unattached path.* In a directed graph, a *pair of twin arcs* is two arcs (*a*,*b*) and (*b*,*a*).

Given a set *X* of vertices, edges, or arcs in a graph *G*, let *G*∖*X* denote the graph obtained by deleting *X* from *G*. When *X* has only one element *x*, we might also write *G*∖*x* instead of *G*∖{*x*}.

### Parameterized Complexity

In the parameterized complexity setting, an instance comes with an integer parameter *k*—formally, a parameterized problem *Q* is a subset of *Σ* ^∗^×ℕ for some finite alphabet *Σ*. We say that the problem is *fixed-parameter tractable* (*FPT*) if there exists an algorithm solving any instance (*x*,*k*) in time *f*(*k*)poly(|*x*|) for some (usually exponential) computable function *f*. It is known that a problem is FPT if and only if it is kernelizable: a kernelization algorithm for a problem *Q* takes an instance (*x*,*k*) and in time polynomial in |*x*|+*k* produces an equivalent instance (*x*′,*k*′) (i.e., (*x*,*k*)∈*Q* if and only if (*x*′,*k*′)∈*Q*) such that |*x*′|+*k*′≤*g*(*k*) for some computable function *g*. The function *g* is the *size of the kernel*, and if it is polynomial, we say that *Q* admits a polynomial kernel.

## Polynomial-Time Solvable Cases

First, we give a simple polynomial time algorithm for the following problem: 
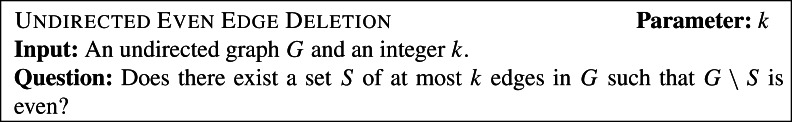


It turns out that this problem is strongly connected to the concept of a *T-join*. If we define *T* to be the set of vertices having odd degree, then Undirected Even Edge Deletion is equivalent with the following classical problem of finding a *T*-join of minimum size: 
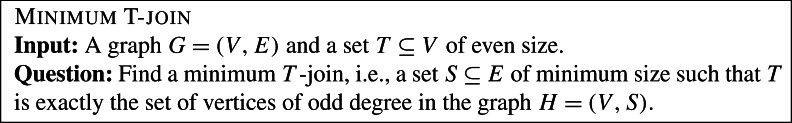


Since Minimum T-join can be solved in cubic time by the algorithm of Edmonds and Johnson [14], we obtain the following consequence:

### Theorem 1

Undirected Even Edge Deletion *can be solved in* *O*(*n* ^3^) *time for an* *n*-*vertex graph*.

Now we turn our attention to the directed version of the problem: 
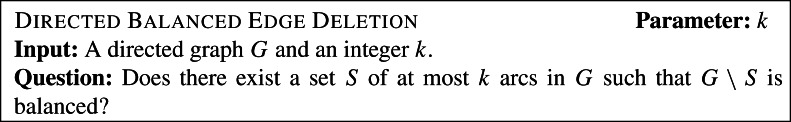


This problem can be formulated as a minimum cost flow problem with unit costs as follows. We create a digraph *G*′ by taking *G* and adding two vertices *s*,*t* (source and sink). Each edge of *E*(*G*) has unit capacity and unit cost. For each vertex *v*∈*V*(*G*) such that *d* ^*in*^(*v*)<*d* ^*out*^(*v*) we add to *G*′ an arc (*s*,*v*) of capacity *d* ^*out*^(*v*)−*d* ^*in*^(*v*) and cost zero. Similarly, for each vertex *v*∈*V*(*G*) such that *d* ^*in*^(*v*)>*d* ^*out*^(*v*) we add to *G*′ an arc (*v*,*t*) of capacity *d* ^*in*^(*v*)−*d* ^*out*^(*v*) and cost zero. Let *f* ^∗^ denote the total capacity of the added arcs (*s*,*v*). In a solvable instance we know that *f* ^∗^≤*k*.

It is straightforward to see that a flow of size *f* ^∗^ and cost at most *k* corresponds to a set *S* of at most *k* arcs for which *G*∖*S* is balanced, and vice versa. Firstly assume that we are given a flow of size *f* ^∗^ and cost at most *k*. As the capacities are integral, the flow is integral as well. Let *S* be the set of arcs of *G* through which a unit flow flows. Clearly, |*S*|≤*k*. As the flow has size *f* ^∗^, for every vertex *v* with *d* ^*in*^(*v*)<*d* ^*out*^(*v*) there is exactly *d* ^*out*^(*v*)−*d* ^*in*^(*v*) flow incoming via the arc from *s*, and for every vertex *v* with *d* ^*in*^(*v*)>*d* ^*out*^(*v*) there is exactly *d* ^*in*^(*v*)−*d* ^*out*^(*v*) flow leaving via the arc to *t*. Hence, every vertex *v* with *d* ^*in*^(*v*)<*d* ^*out*^(*v*) has exactly *d* ^*out*^(*v*)−*d* ^*in*^(*v*) more outgoing arcs from *S* than incoming arcs from *S*, every vertex *v* with *d* ^*in*^(*v*)>*d* ^*out*^(*v*) has exactly *d* ^*in*^(*v*)−*d* ^*out*^(*v*) more incoming arcs from *S* than outgoing arcs from *S*, while for all the vertices with *d* ^*in*^(*v*)=*d* ^*out*^(*v*) the numbers of incoming and outgoing arcs from *S* are equal. This implies that if we remove all the arcs of *S* from the graph, we end with a balanced graph. On the other hand, if *S* is such that *G*∖*S* is balanced, then setting unit flow on *S*, zero flow on the other arcs of *G*, and maximum flow on arcs adjacent to the source and the sink yields a correct flow between *s* and *t* of size *f* ^∗^ and cost |*S*|.

Therefore, in order to find a solution of minimum size it suffices to find a minimum cost flow of size *f* ^∗^. As *f* ^∗^≤*k* and each arc has unit cost, this can be done in *O*(*nm*log*n*loglog*k*) time [1], where *n*=|*V*(*G*)| and *m*=|*E*(*G*)|. Note that the above argument also handles an annotated case, where we require that *S*⊆*E*_*a*_ for a set *E*_*a*_⊆*E* given in the input, as we can put zero capacities on *E*∖*E*_*a*_. This yields the following:

### Theorem 2

Directed Balanced Edge Deletion *can be solved in time complexity* *O*(*nm*log*n*loglog*k*) *for an input graph with* *n* *vertices and* *m* *edges*, *even in an annotated case where some edges are forbidden to delete*.

## Eulerian Edge-Deletion Problems

In this section we examine the following problems: 
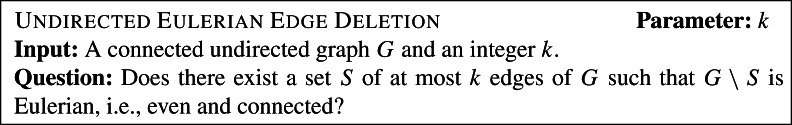



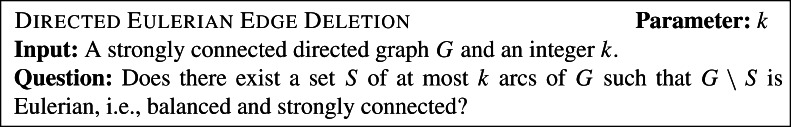


The undirected problem can be easily seen to be NP-hard by observing that a cubic graph contains a Hamiltonian cycle if and only if it can be made Eulerian by edge deletions. Indeed, if deleting a set of edges from a cubic graph *G* results in an Eulerian graph *G*′, then each vertex in *G*′ must have degree 2, so *G*′ must be a Hamiltonian cycle of *G*. Since the Hamiltonian Cycle problem restricted to cubic planar graphs is NP-hard [16] the result follows. The directed version can be treated in a similar way using NP-hardness from [30].

### Theorem 3

*The* Undirected *and* Directed Eulerian Edge Deletion *problems are NP*-*hard*, *even when restricted to inputs* (*G*,*k*) *where* *G* *is a planar* (*directed*) *graph with maximum degree at most* 3, *and* *k*=|*E*(*G*)|.

In Sect. 4.1, we show that both versions of the problem are FPT and can be solved in time 2^*O*(*k*log*k*)^ *n* ^*O*(1)^. The algorithm is based on a novel randomized selection argument. In Sect. 4.3, we sharpen Theorem 3 by showing that the problems do not admit a polynomial kernel. In some sense, the nonexistence of polynomial kernels suggests that randomized selection or a similar technique is inherently required for the problems, as they cannot be solved by simple reduction rules.

### FPT Algorithms

We have seen in Sect. 3 that removing edges to make all the vertices even can be expressed as a *T*-join problem, where *T* is the set of odd vertices. Thus Undirected Eulerian Edge Deletion requires us to find a *T*-join *S* such that *G*∖*S* is connected. Observe that if *G* is connected, and *G*∖*S* has a connected subgraph *W* containing the endpoints of every edge in *S*, then *G*∖*S* is connected as well. We will call such a subgraph *W* a *witness* of *S*. Therefore, the right way to look at the problem is that we need to find a pair (*S*,*W*), where is *S* is a *T*-join and *W* is the witness of *S*. It is clear that the problem has a solution if and only if such a pair exists.

Our approach for finding a pair (*S*,*W*) is the following. We randomly color the edges of the graph red and blue, and try to find a pair (*S*,*W*) where *S* uses only red edges and the subgraph *W* uses only blue edges. We would like to ensure that if a suitable pair (*S*,*W*) exists, then it is correctly colored red and blue with probability at least 2^−*O*(*k*log*k*)^. However, in general the size of *W* can be very large (unbounded in *k*; an example is provided in Sect. 4.2) and therefore the probability of a correct coloring can be very small. We get around this problem by observing that edges “far” from *T* can be always colored blue, and there is a witness *W* that uses only a bounded number of edges “close” to *T*. Formally, we say that an edge *e* is *close* if at least one endpoint of *e* is at distance at most *k* from *T*; otherwise, *e* is *far*. The following two lemmas contain the crucial combinatorial ideas of the algorithm:

#### Lemma 1

*If* *S* *is an optimum solution of size at most* *k*, *then each edge of* *S* *is close*.

#### Proof

As removing a cycle from *S* would still yield a solution, *H*=(*V*,*S*) has to be a forest for an optimum solution *S*. Each connected component of *H* that is not an isolated vertex contains a vertex from *T*, as each tree contains vertices of odd degree (for example, leaves). Since |*S*|≤*k*, each vertex in such a connected component is at distance at most *k* from *T*, and thus each edge in *S* is close. □

#### Lemma 2

*If* *S* *is an optimum solution of size at most* *k*, *then* *S* *has a witness* *W* *having at most* (2*k*−1)(2*k*+2) *close edges*.

#### Proof

Let *X* be the set of endpoints of the edges in *S*. Note that *T*⊆*X* and |*X*|≤2|*S*|≤2*k*. Let *i* be the smallest integer such that *G*∖*S* has a subgraph *W* containing *X*, having exactly *i* connected components and at most (|*X*|−*i*)(2*k*+2) close edges (such *i* and *W* always exist as for *i*=|*X*| we can take *W*=(*X*,∅)). If *i*=1, then we are done. Otherwise, we can assume that each component of *W* contains a vertex of *X*; let *P* be a shortest path in *G*∖*S* that connects two different components of *W*. Denote these components *K*_1_ and *K*_2_.

We claim that only the first *k*+1 and the last *k*+1 edges of *P* may be close. If this is true, then adding *P* to *W* decreases the number of components and increases the number of close edges by at most 2*k*+2, contradicting the minimality of *i*.

Suppose that an edge *e* is close, but it is not among the first or last *k*+1 edges, i.e., both of its endpoints are at distance greater than *k* from both *K*_1_ and *K*_2_ on *P*. As *e* is close, it has an endpoint *v* such that there is a path *P*′ of length at most *k* connecting *v* and *T*. As *T*⊆*X*, the path *P*′ connects *v* to a component *K*′ of *W*. Assuming without loss of generality that *K*′≠*K*_1_, the concatenation of *P*′ and the subpath of *P* between *K*_1_ and *v* is a walk *P*″ connecting two different components of *W*. As the distance of *v* from *K*_2_ on *P* is more than *k*, the walk *P*″ is shorter than *P*, contradicting the minimality of *P*. □

We observe that even though the number of close edges in the witness can be bounded polynomially in *k*, the whole witness can be arbitrarily large. An example of such a situation is described in Sect. 4.2.

Now, we are ready to state our algorithm, working as follows:

1. Determine which edges are close and which are far.
2. Make each close edge independently with probability 1/*k* ^2^ red; every edge that is not red becomes blue.
3. If there is more than one connected component of the blue edges containing a vertex from *T*, return NO; otherwise let *K*_*B*_ be this unique component.
4. Solve Minimum T-join instance (*G*_*R*_,*T*), where *G*_*R*_ is the graph induced by the red edges with both endpoints in *K*_*B*_. If the solution is of size at most *k*, return it, otherwise return NO.

#### Lemma 3

*If the algorithm returns a solution* *S*, *then* *S* *is a proper solution to* Undirected Eulerian Edge Deletion.

#### Proof

By the definition of Minimum T-join, *G*∖*S* is even. The component *K*_*B*_ of blue edges ensures that the endpoints of *S* are in the same component of *G*∖*S*, i.e., *G*∖*S* is connected. □

#### Lemma 4

*If the* Undirected Eulerian Edge Deletion *instance* (*G*,*k*) *was a YES*-*instance*, *the algorithm returns a solution with probability at least* 1/2^*O*(*k*log*k*)^.

#### Proof

Let *S* be an optimum solution to (*G*,*k*), and let *W* be a witness having at most (2*k*−1)(2*k*+2) close edges, guaranteed by Lemma 2. In the algorithm:

1. With probability at least (1/*k* ^2^)^*k*^=1/2^2*k*log*k*^ each edge of *S* becomes red.
2. With probability at least (1−1/*k* ^2^)^(2*k*−1)(2*k*+2)^=*Ω*(1) each close edge of *W* becomes blue (and hence every edge of *W* is blue).

The above events are independent, since *S* and *W* do not share edges. Furthermore, if both events happen, then *W* will connect all the endpoints of the edges from *S*. Therefore, all of these endpoints will be contained in one connected component *K*_*B*_ of the graph induced by blue edges, which in particular connects all the vertices from *T*. Thus, with probability 1/2^*O*(*k*log*k*)^, every edge of *S* appears in *G*_*R*_ in the last step of the algorithm and the Minimum T-join instance has a solution of size at most *k*. □

#### Theorem 4

*Both the* Undirected *and* Directed Eulerian Edge Deletion *problems are fixed*-*parameter tractable with parameter* *k*.

#### Proof

By Lemmas 3 and 4, the presented algorithm for Undirected Eulerian Edge Deletion finds a solution with probability 1/2^*O*(*k*log*k*)^, and never produces a wrong output, that is removal of the returned set of edges always makes the graph Eulerian. Since the algorithm runs in *O*(*n* ^3^) time for an *n*-vertex graph, we immediately obtain a randomized FPT Monte-Carlo algorithm, running in 2^*O*(*k*log*k*)^ *n* ^3^ time.

We present how to derandomize the described algorithm using the standard technique of splitters. An (*m*,*r*,*r* ^2^)-*splitter* is a family of functions from {1,2,…,*m*} to {1,2,…,*r* ^2^}, such that for any subset *X*⊆{1,2,…,*m*} of size *r*, one of the functions in the family is injective on *X*. Naor et al. [27] gave an explicit construction of an (*m*,*r*,*r* ^2^)-splitter of size *O*(*r* ^6^log*r*log*m*).

In Step 3 of the algorithm we want to separate the solution *S* (of size at most *k*) from the set of close edges of the witness *W* (of size at most *ℓ*=(2*k*−1)(2*k*+2)). Let *m* be the cardinality of the set of close edges in the graph, we may identify {1,2,…,*m*} with this set. Instead of the random coloring process, we can try every function *f* in a (*m*,*k*+*ℓ*,(*k*+*ℓ*)^2^)-splitter and every set *F*⊆{1,2,…,(*k*+*ℓ*)^2^} of size *k*. For a particular choice of *f* and *F*, we color red those close edges *e* for which *f*(*e*)∈*F*. By the definition of the splitter, if there exists a solution *S* with a witness *W*, there will be a function *f* that is injective on the set of close edges of *S*∪*W* and a subset *F* such that *f*(*e*)∈*F* if *e*∈*S* and *f*(*e*)∉*F* if *e* is a close edge in *W*. Note that the size of (*m*,*k*+*ℓ*,(*k*+*ℓ*)^2^)-splitter is bounded polynomially in the input size, whereas there are 2^*O*(*k*log*k*)^ choices for the set *F*.

Regarding Directed Eulerian Edge Deletion, we can use a slightly modified version of our randomized algorithm, which then can be derandomized in exactly the same manner. After defining the set *T* of terminals to contain the unbalanced vertices, we forget about the orientation of the arcs, and perform Steps 1–3 of the algorithm. We adjust Step 4 by solving an annotated Directed Balanced Edge Deletion instance (*G*,*k*) where only red arcs can be deleted. Observe that this algorithm in fact looks for a set of edges *S* of size at most *k* such that *G*∖*S* is balanced and weakly connected. However, every graph that is weakly connected and balanced is Eulerian, thus the algorithm returns the solution to Directed Balanced Edge Deletion with high probability, if one exists. □

### An Example of a Large Witness Set *W*

In this section we give a simple example that the witness graph *W*, considered in the FPT algorithms in Sect. 4.1, may be arbitrarily large and may contain *Ω*(*k* ^2^) close edges. This lower bound on the number of close edges matches the upper bound given by Lemma 2. We construct a graph *G* as follows. First take a cycle of length 2*kM* for some *M*>2*k* and let *v*_0_,*v*_1_,…,*v*_2*k*−1_ be a sequence of evenly distributed vertices on this cycle, i.e., *v*_*i*_=*w*_*iM*_, where *w*_0_,*w*_1_,…,*w*_2*kM*−1_ are vertices of the cycle, lying in this order. Moreover, for each 0≤*i*<*k* we connect the vertices *v*_2*i*_ and *v*_2*i*+1_. Note that *S*={*v*_2*i*_ *v*_2*i*+1_:0≤*i*<*k*} is the only feasible solution of size *k* to the Undirected Eulerian Edge Deletion problem in the graph *G*, but any witness *W* of *S* needs to contain a path of length (2*k*−1)*M*. Moreover, such a path contains roughly 2*k*(2*k*−1)=*Ω*(*k* ^2^) close edges.

Note that in the above construction it is not crucial to start from a long cycle, as any Eulerian graph of large diameter would suffice. In such a graph we simply take the vertices {*v*_*i*_:0≤*i*<2*k*} to be any set of vertices that are pairwise distant.

### Non-existence of a Polynomial Kernel for Undirected and Directed Eulerian Edge Deletion

The aim of this subsection is to prove the following theorem.

#### Theorem 5

*If* $\textrm {NP} \not \subseteq \textrm {coNP}/\textrm {poly}$, *then there is no polynomial kernel for the* Undirected *and* Directed Eulerian Edge Deletion *problems with parameter* *k*, *even if the input graph has maximum degree at most* 4.

We use the cross-composition technique introduced by Bodlaender et al. [5]. Let us recall the crucial definitions.

#### Definition 1

(Polynomial equivalence relation [5])

An equivalence relation $\mathcal{R}$ on *Σ* ^∗^ is called a *polynomial equivalence relation* if (1) there is an algorithm that given two strings *x*,*y*∈*Σ* ^∗^ decides whether $\mathcal{R}(x,y)$ in (|*x*|+|*y*|)^*O*(1)^ time; (2) for any finite set *S*⊆*Σ* ^∗^ the equivalence relation $\mathcal{R}$ partitions the elements of *S* into at most (max_*x*∈*S*_|*x*|)^*O*(1)^ classes.

#### Definition 2

(Cross-composition [5])

Let *L*⊆*Σ* ^∗^ and let *Q*⊆*Σ* ^∗^×ℕ be a parameterized problem. We say that *L* *cross-composes* into *Q* if there is a polynomial equivalence relation $\mathcal{R}$ and an algorithm which, given *p* strings *x*_1_,*x*_2_,…,*x*_*p*_ belonging to the same equivalence class of $\mathcal{R}$, computes an instance (*x* ^∗^,*k* ^∗^)∈*Σ* ^∗^×ℕ in time polynomial in $\sum_{i=1}^{p} |x_{i}|$ such that (1) (*x* ^∗^,*k* ^∗^)∈*Q* if and only if *x*_*i*_∈*L* for some 1≤*i*≤*p*; (2) *k* ^∗^ is bounded polynomially in $\max_{i=1}^{p} |x_{i}| + \log p$.

#### Theorem 6

([5], Theorem 9)

*If* *L*⊆*Σ* ^∗^ *is NP*-*hard under Karp reductions and* *L* *cross*-*composes into the parameterized problem* *Q* *that has a polynomial kernel*, *then* NP⊆coNP/poly.

We apply Theorem 6 on the following language *L*: 
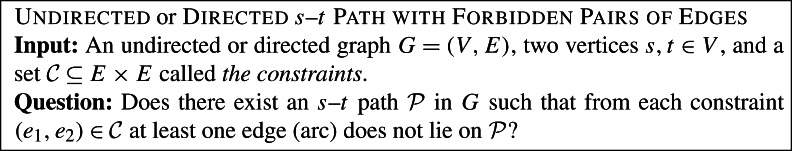


The undirected version of this problem with forbidden pairs of vertices was proven to be NP-hard by Kolman and Pangrác [19] and their proof can be easily modified to handle our case as well.

#### Lemma 5

Undirected *and* Directed *s–t* Path with Forbidden Pairs of Edges *are NP*-*hard under Karp reductions*, *even in the case where each vertex has maximum degree three*, *s* *and* *t* *have degree one*, *and*, *in the directed case*, *each vertex has maximum in*- *and outdegree two*.

#### Proof

We first provide a Karp reduction from the Clique problem to our problems without the degree condition. Let (*H*,*k*) be a Clique instance. We construct an Undirected or Directed *s*–*t* Path with Forbidden Pairs of Edges instance $(G,s,t,\mathcal{C})$ as follows. To construct the graph *G*, we start by adding vertices *s*, *t* and *p*_*i*_ for 0≤*i*≤*k* and edges *sp*_0_ and *p*_*k*_ *t* (arcs (*s*,*p*_0_) and (*p*_*k*_,*t*)). Then for each 1≤*i*≤*k* and *v*∈*V*(*H*) we introduce a vertex $x_{i}^{v}$ and edges $p_{i-1}x_{i}^{v}$ and $x_{i}^{v}p_{i}$ (arcs $(p_{i-1},x_{i}^{v})$ and $(x_{i}^{v},p_{i})$). Finally, for each 1≤*i*,*j*≤*k* and *u*,*v*∈*V*(*H*) such that *uv*∉*E*(*H*) (possibly *u*=*v*), we introduce constraints $(x_{i}^{u}p_{i},x_{j}^{v}p_{j})$ and $(x_{j}^{u}p_{j},x_{i}^{v}p_{i})$.

Let us now verify the correctness of the above reduction. If {*v*_1_,*v*_2_,…,*v*_*k*_}⊆*V*(*H*) is a vertex set of a *k*-clique in *H*, then a path consisting of edges (or corresponding arcs) *sp*_0_, *p*_*k*_ *t* and $p_{i-1}x_{i}^{v_{i}}$, $x_{i}^{v_{i}}p_{i}$ for 1≤*i*≤*k* is a feasible solution to the instance $(G,s,t,\mathcal{C})$. In the other direction, note that any simple path from *s* to *t* visits for each 1≤*i*≤*k* exactly one vertex from the set $\{x_{i}^{v}: v \in V(H)\}$, say $x_{i}^{v_{i}}$. We claim that {*v*_1_,*v*_2_,…,*v*_*k*_} induces a *k*-clique in *H*. To see this note that the introduced constraints imply that if *i*≠*j* then *v*_*i*_≠*v*_*j*_ and *v*_*i*_ *v*_*j*_∈*E*(*H*).

To obtain the degree bounds, note that each vertex *v*∈*V*(*G*) with *d*_*G*_(*v*)≥3 can be replaced with a (directed) cycle of length *d*_*G*_(*v*), where each edge (arc) previously incident to *v* is now connected to a different vertex on the cycle. □

To finish the proof of Theorem 5 we need to show a cross-composition algorithm. This is done in the following lemma.

#### Lemma 6

Undirected (Directed) *s–t* Path with Forbidden Pairs of Edges *cross*-*composes to* Undirected (Directed) Eulerian Edge Deletion. *If the input instances have degrees bounded as in Lemma* 5 *then the output instance can be made to have maximum degree* 4.

#### Proof

For the equivalence relation $\mathcal{R}$ we take an almost trivial relation that sorts all malformed instances into one equivalence class and all well-formed into another one. If we are given malformed instances, we simply output a trivial NO-instance. Thus in the rest of the proof we assume we are given a sequence $(G_{i},s_{i},t_{i},\mathcal{C}_{i})_{i=1}^{p}$ of Undirected or Directed *s*–*t* Path with Forbidden Pairs of Edges instances.

We now construct an Undirected or Directed Eulerian Edge Deletion instance (*G*,*k*). We start by obtaining a graph $G_{i}'$ for each 1≤*i*≤*p* as follows. First we subdivide each edge *e*∈*E*(*G*_*i*_) with new vertices $x_{e}^{C}$, one for each constraint $C \in\nobreak \mathcal{C}_{i}$ that contains *e*. Then for each constraint $C=(e_{1},e_{2}) \in \mathcal{C}_{i}$ we introduce vertices $z_{1}^{C}$ and $z_{2}^{C}$ and create a (directed) cycle $x_{e_{1}}^{C}, z_{1}^{C}, x_{e_{2}}^{C}, z_{2}^{C}$. By *V*(*G*_*i*_) we denote the subset of $V(G_{i}')$ containing vertices different than $x_{e}^{C}$ and $z_{\alpha}^{C}$. To construct the graph *G*, we first take the union of all graphs $G_{i}'$ and identify all vertices *s*_*i*_ into one vertex *s* ^∗^ and all vertices *t*_*i*_ into one vertex *t* ^∗^. Let $V^{0} = \{s^{*},t^{*}\} \cup \bigcup_{i=1}^{p} V(G_{i}) \setminus \{s_{i},t_{i}\}$. Second, we introduce a new vertex *r* and connect it to the rest of the graph as follows. In the undirected case for each *v*∈*V* ^0^∖{*s* ^∗^,*t* ^∗^} we connect *r* and *v* with one or two unattached paths of length 2, so that in *G* the vertex *v* is even. In the directed case, we connect *r* and *v* with some positive number of unattached directed paths of length 2, so that in *G* the vertex *v* is balanced. We do almost the same construction to connect *s* ^∗^ and *t* ^∗^ to *r*, but we ensure that the degrees of *s* ^∗^ and *t* ^∗^ are odd (in the undirected case) or that $d_{G}^{in}(s^{*}) + 1 = d_{G}^{out}(s^{*})$ and $d_{G}^{in}(t^{*}) = d_{G}^{out}(t^{*}) + 1$ (in the directed case). Note that *r* is even (balanced). Finally, we set $k = \max_{i=1}^{p} |V(G_{i}')|-1 = O(\max_{i=1}^{p} |V(G_{i})| + |\mathcal{C}_{i}|)$.

It is clear that the above construction can be done in polynomial time and that the parameter *k* is bounded polynomially in the maximum size of the input instances. We now verify the correctness of the construction, i.e., (*G*,*k*) is a YES-instance of Undirected or Directed Eulerian Edge Deletion if and only if at least one of the instances $(G_{i},s_{i},t_{i},\mathcal{C}_{i})_{i=1}^{p}$ of Undirected or Directed *s*–*t* Path with Forbidden Pairs of Edges is a YES-instance. Then, we discuss how we can modify the construction so that all the vertices of the resulting graph have degree bounded by 4.

#### Correctness

First, let $\mathcal{P}$ be a simple path that is a feasible solution to $(G_{j},s_{j},t_{j},\mathcal{C}_{j})$ for some 1≤*j*≤*p*. The path $\mathcal{P}$ naturally defines a simple path $\mathcal{P}'$ in $G_{j}'$ and in *G*. We claim that the edge set *S* of $\mathcal{P}'$ is a feasible solution to the constructed Directed or Undirected Eulerian Edge Deletion instance. As it is contained in $G_{j}'$, we have |*S*|≤*k*. Since in *G* the only odd (unbalanced) vertices were *s* ^∗^ and *t* ^∗^, *G*∖*S* is even (balanced). We now verify that each vertex *v*∈*V*(*G*) is (weakly) connected to the vertex *r* in *G*∖*S*. It is clear for each *v*∈*V* ^0^, since *E*_*r*_∩*S*=∅, where *E*_*r*_ denotes the set of edges in the paths between *V* ^0^ and *r* (note that each *v*∈*V* ^0^ is connected to *r* by a positive number of paths). For the other vertices, note that if $C = (e_{1},e_{2}) \in \bigcup_{i=1}^{p} \mathcal{C}_{i}$, then either *e*_1_ or *e*_2_ does not belong to $\mathcal{P}$ (say $e_{1} \notin \mathcal{P}$) and the cycle $x_{e_{1}}^{C}, z_{1}^{C}, x_{e_{2}}^{C}, z_{2}^{C}$ is connected to *r* via subdivided edge *e*_1_ and its endpoints. Note that in the directed case it is sufficient to ensure only weak connectivity, as a balanced graph is weakly connected if and only if it is strongly connected.

In the opposite direction, let *S* be a solution to the constructed Directed or Undirected Eulerian Edge Deletion instance. We assume that |*S*| is minimum possible. It is easy to see that since *G*∖*S* is even (balanced) and |*S*| is minimal, *S* needs to induce a simple path $\mathcal{P}'$ from *s* ^∗^ to *t* ^∗^. This path cannot contain a neighbor *r*′ of *r*, since otherwise *r*′ becomes isolated in *G*∖*S*. Thus $\mathcal{P}'$ is contained in graph $G_{j}'$ for some 1≤*j*≤*p*. Moreover, note that $\mathcal{P}'$ cannot contain any vertex $z_{\alpha}^{C}$, as otherwise $z_{\alpha}^{C}$ becomes isolated in *G*∖*S*. Thus $\mathcal{P}'$ naturally defines a path $\mathcal{P}$ in *G*_*j*_ with endpoints *s*_*j*_ and *t*_*j*_. Observe that if for some $C = (e_{1},e_{2}) \in \mathcal{C}_{j}$ the path $\mathcal{P}$ contained both *e*_1_ and *e*_2_, then in *G*∖*S* the cycle $x_{e_{1}}^{C}, z_{1}^{C}, x_{e_{2}}^{C}, z_{2}^{C}$ would be unreachable from the rest of the graph *G*. Thus, $\mathcal{P}$ is a feasible solution to the instance $(G_{j},s_{j},t_{j},\mathcal{C}_{j})$.

#### Degree Reduction

We now show how to modify the presented construction to obtain an instance with maximum degree 4. First note that if *v*∈*V*(*G*)∖(*V* ^0^∪{*r*}) we clearly have *d*_*G*_(*v*)≤4. Moreover, if the degrees in $(G_{i},s_{i},t_{i},\mathcal{C}_{i})$ are bounded as in Lemma 5, then for any *v*∈*V* ^0^∖{*s* ^∗^,*t* ^∗^} the number of unattached paths connecting *v* and *r* can be chosen so that *v* is even (balanced) and we have *d*_*G*_(*v*)≤4 in the undirected case and $d_{G}^{in}(v), d_{G}^{out}(v) \leq 2$ in the directed one. Thus, we are left with the vertices *s* ^∗^, *t* ^∗^ and *r*.

We first reduce the degree of vertices *s* ^∗^ and *t* ^∗^. By duplicating some input instances we may ensure that their number is a power of two, *p*=2^*ℓ*^. We replace *s* ^∗^ and *t* ^∗^ with full binary trees *T*_*s*_ and *T*_*t*_ of height *ℓ*, rooted at *s* ^*r*^ and *t* ^*r*^. In the directed case, the edges in the tree *T*_*s*_ are directed towards the leaves, whereas the edges in the tree *T*_*t*_ are directed towards the root *t* ^*r*^. For each instance $(G_{j}, s_{j}, t_{j}, \mathcal{C}_{j})$ we identify *s*_*j*_ with one leaf in *T*_*s*_ and *t*_*j*_ with one leaf in *T*_*t*_, so that each instance is assigned to different leaves in *T*_*s*_ and *T*_*t*_. Finally, we connect each vertex of the trees *T*_*s*_ and *T*_*t*_ with *r* using one or two unattached paths of length two, so that *d*_*G*_(*s* ^*r*^)=*d*_*G*_(*t* ^*r*^)=3 ($d_{G}^{out}(s^{r}) = 2 = 1 +d_{G}^{in}(s^{r})$ and $d_{G}^{in}(t^{r}) = 2 = 1 + d_{G}^{out}(t^{r})$ in the directed case) and each other vertex in *T*_*s*_ and *T*_*t*_ is of degree 4 and, in the directed case, balanced.

As for the vertex *r*, we replace it with a (directed) cycle of length *d*_*G*_(*r*)/2, with each vertex on the cycle adjacent to exactly two edges previously incident to *r* (one incoming arc and one outgoing arc in the directed case). Finally, we set $k = 2\ell + \max_{i=1}^{p} |V(G_{i}')|-1 = O(\log p + \max_{i=1}^{p} |V(G_{i})| + |\mathcal{C}_{i}|)$. Note that now a minimum solution *S* to the constructed Directed or Undirected Eulerian Edge Deletion instance needs to induce a simple path $\mathcal{P}'$ from *s* ^*r*^ to *t* ^*r*^ that first goes down the tree *T*_*s*_ to a leaf *s*_*j*_ (for some 1≤*j*≤*p*), then traverses the graph $G_{j}'$, inducing a solution $\mathcal{P}$ to the instance $(G_{j}, s_{j}, t_{j}, \mathcal{C}_{j})$, and finally goes up the tree *T*_*t*_ starting at the leaf *t*_*j*_.  □

## Node-Deletion Problems

We first consider the following two node-deletion problems: 
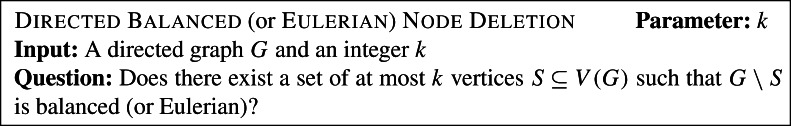


The undirected versions of these problems, namely Undirected Even and Undirected Eulerian Node Deletion, are defined analogously. While these undirected variants were already shown to be W[1]-hard with parameter *k* by Cai and Yang [9], the complexity of the directed versions has not been studied yet. The following theorem shows that they are intractable as well.

### Theorem 7

Directed Balanced Node Deletion *and* Directed Eulerian Node Deletion *are* NP-*hard and* W[1]-*hard with parameter* *k*.

### Proof

We firstly treat the balanced case, then we proceed to the Eulerian case.

### Balanced Case

We present an FPT-reduction from the Disjoint Set Cover problem to Directed Balanced Node Deletion. The input of this problem is a triple $(U,\mathcal{F},k)$ where *U* is some universe, $\mathcal{F}=\{F_{1}, \dots, F_{n}\}$ is a family of subsets of *U*, and *k* is an integer. The task is to decide whether there is a collection $\mathcal{H} \subseteq \mathcal{F}$ with $|\mathcal{H}|\leq k$ that covers each element of *U* exactly once, i.e., such that the sets in $\mathcal{H}$ are pairwise disjoint and their union is *U*. Given such an input, we are going to construct a directed graph *G* such that (*G*,*k*) is a YES-instance of Directed Balanced Node Deletion if and only if $(U,\mathcal{F},k)$ is a YES-instance of Disjoint Set Cover. Moreover, the presented reduction will be polynomial-time computable. As Disjoint Set Cover is NP-hard, and also W[1]-hard with parameter *k* [25], this suffices to prove the theorem.

Given $(U,\mathcal{F},k)$, for any *u*∈*U*, let *n*(*u*) denote the number of sets in $\mathcal{F}$ that contain *u*. For each *u*∈*U*, we introduce two vertices *u* ^1^ and *u* ^2^ in *G*, and connect them by *n*(*u*)−1 unattached paths of length *k*+2, each starting from *u* ^1^ and leading to *u* ^2^. We denote by *D* the set of all internal vertices on these paths, each having degree 2 in *G*. Furthermore, for each $F_{i} \in \mathcal{F}$ we introduce a vertex *f*_*i*_, and add the arcs (*f*_*i*_,*u* ^1^) and (*u* ^2^,*f*_*i*_) for each *u*∈*F*_*i*_. This finishes the construction of *G*. It is not hard to see that the reduction is indeed polynomial.

Now, suppose that *G*∖*S* is balanced for some *S*⊆*V*(*G*), |*S*|≤*k*. Note that if *S* contains any vertex *d* on a path of length *k*+2 leading from some *u* ^1^ to *u* ^2^ (allowing *d*=*u* ^1^ or *d*=*u* ^2^), then all *k*+1 internal vertices of this path should be in *S*, which contradicts |*S*|≤*k*. Thus, we get that $S \subseteq \{f_{i} \mid F_{i} \in \mathcal{F} \}$. Observe also that for each *u*∈*U*, we get $d_{G}^{in}(u^{1})=d_{G}^{out}(u^{1})+1=d_{G \setminus S}^{out}(u^{1})+1$, hence the deletion of *S* must decrease the indegree of each vertex *u* ^1^ (*u*∈*U*) by exactly one. By the definition of *G*, this means that the sets *F*_*i*_ for *f*_*i*_∈*S* form a family of at most *k* pairwise disjoint sets together covering *U*, as required.

For the other direction, it is straightforward to see that if $\mathcal{S} \subseteq \mathcal{F}$ is a solution for the Disjoint Set Cover instance, then deleting the vertex set $\{ f_{i} \mid F_{i} \in \mathcal{S}\}$ from *G* results in a balanced graph. This observation relies on the fact that $d_{G}^{in}(u^{1})=d_{G}^{out}(u^{1})+1$ and $d_{G}^{in}(u^{2})=d_{G}^{out}(u^{2})-1$ hold for each *u*∈*U*, and that *d* ^*in*^(*v*)=*d* ^*out*^(*v*) holds for each remaining vertex *v*. This proves our statement for Directed Balanced Node Deletion.

### Eulerian Case

Now, we give a reduction from Directed Balanced Node Deletion problem to Directed Eulerian Node Deletion. Given an input (*G*,*k*) we construct (*G*′,*k*) in polynomial time such that there is a set *S*⊆*V*(*G*) with |*S*|≤*k* for which *G*∖*S* is balanced if and only if there is a set *S*′⊆*V*(*G*′) with |*S*′|≤*k* for which *G*′∖*S*′ is Eulerian.

To construct *G*′, we simply add to *G* a new vertex *r*, and connect each vertex to *r* by a pair of twin arcs. On one hand, if *G*′∖*S*′ is Eulerian for some *S*′⊆*V*(*G*′), then *G*∖(*S*′∖{*r*}) must be balanced, as deleting *r* from the balanced graph *G*′∖*S*′ still yields a balanced graph. On the other hand, if *G*∖*S* is balanced for some *S*⊆*V*(*G*), then observe that *G*′∖*S* is balanced as well. Furthermore, since each vertex in *G*′∖*S* is connected by a pair of twin arcs to *r*, *G*′∖*S* is Eulerian as well, finishing the proof.  □

As Table 1 shows, the node-deletion variant is W[1]-hard in all four cases, while the edge-deletion version is FPT or even polynomial-time solvable. What makes the node-deletion versions harder? One obvious difference is that in the edge-deletion problem the answer is trivially no if there are more than 2*k* odd/unbalanced vertices, but the node-deletion versions can have a solution even if the number of such nodes is unbounded. This suggests that the higher complexity comes from the ability of affecting the degree of many vertices by a single vertex deletion. Indeed, if every vertex has degree bounded by *Δ*, then we can solve all of the above defined node-deletion problems in *O*((*Δ*+1)^*k*^(|*V*(*G*)|+|*E*(*G*)|)) time by a simple branching algorithm. However, this interpretation is not fully correct: as we shall show, the node-deletion problems are hard even if we are allowed to delete only vertices of constant degree.

To this end, we define the following variation of the four different node-deletion problems, where *α* can be Undirected Even, Undirected Eulerian, Directed Balanced, or Directed Eulerian: 
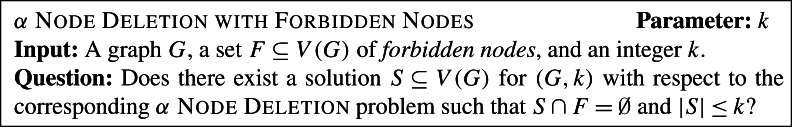


In other words, we require the solution to be disjoint from a set of *forbidden vertices*. A vertex is *allowed*, if it is not forbidden. For each of the four node-deletion problems, the above variant is at least as hard as the original problem, and in fact has the same complexity: this variant can easily be reduced to the original version, by attaching long unattached cycles to every forbidden vertex. Furthermore, we show that allowing only the deletion of bounded-degree vertices does not make the problem easier:

### Theorem 8

*Each of the problems* *α* Node Deletion with Forbidden Nodes *where* *α* *is* Undirected Even, Undirected Eulerian, Directed Balanced, *or* Directed Eulerian *remains* W[1]-*hard with parameter* *k*, *even if each allowed vertex has degree at most* 4.

We prove Theorem 8 in two steps. Theorem 9 deals with the cases where we aim for an even or a balanced graph, while Theorem 10 handles the two Eulerian cases. Let *Δ*_*a*_ be the maximum degree taken over all allowed vertices.

### Theorem 9

Undirected Even *and* Directed Balanced Node Deletion with Forbidden Nodes *remain* W[1]-*hard with parameter* *k*, *even if* *Δ*_*a*_=4.

### Proof

We firstly describe the undirected case, then we present how the construction can be refined for the directed case.

### Undirected Case

We present a parameterized reduction from the W[1]-hard Multicoloured Clique problem [15] to Undirected Even Node Deletion with Forbidden Nodes with *Δ*_*a*_=4. The input of Multicoloured Clique is a graph *G*=(*V*,*E*) and an integer *k* together with a partition *V*_1_,*V*_2_,…,*V*_*k*_ of *V* where each *V*_*i*_ is an independent set; the task is to find a clique of size *k* in *G*. W.l.o.g. we assume that *k*≥3, as otherwise the instance can be solved via brute-force.

Firstly, we claim that one can assume that for each vertex *v* and for each *V*_*j*_ it holds that |*N*(*v*)∩*V*_*j*_| is even. This can be achieved without changing the answer to our instance in the following manner. For each pair *i*,*j* (1≤*i*,*j*≤*k*, *i*≠*j*) we introduce a new vertex *a* ^*i*,*j*^ into *V*_*j*_ and connect it with all the vertices of *V*_*i*_ having an odd number of neighbors in *V*_*j*_. Consider the graph induced by *V*_*i*_∪*V*_*j*_∪{*a*_*i*,*j*_,*a*_*j*,*i*_}. By the definition of *a*_*i*,*j*_,*a*_*j*,*i*_ we know that all the vertices of *V*_*i*_ and *V*_*j*_ have even degrees in this graph. As the sum of degrees in every graph is even, *a*_*i*,*j*_ has odd degree if and only if *a*_*j*,*i*_ has. Therefore, we introduce an edge *a*_*i*,*j*_ *a*_*j*,*i*_ if the degree of *a*_*i*,*j*_ is odd. From the construction we infer that the claimed property holds, i.e., for each vertex *v* and for each *V*_*j*_ we have that |*N*(*v*)∩*V*_*j*_| is even. Note that vertex *a* ^*i*,*j*^ cannot be contained in any *k*-clique in *G*, as *N*(*a* ^*i*,*j*^)⊆*V*_*i*_ (recall that *a* ^*j*,*i*^ has been introduced into *V*_*i*_). This ensures the correctness of the construction.

**Fig. 1:**
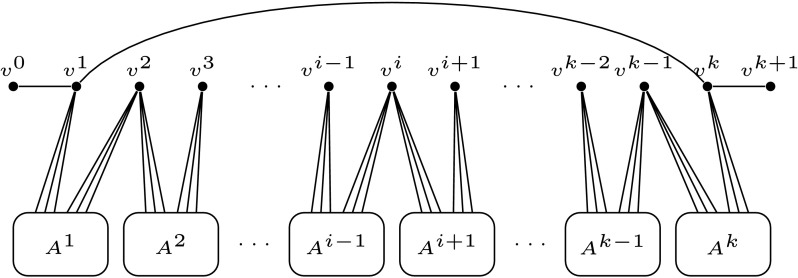
Vertex gadget *G*_*v*_ for *v*∈*V*_*i*_

We are going to construct an instance (*G*′,*F*,*k*′) of Undirected Even Node Deletion with Forbidden Nodes with *k*′=*k* ^2^+*k*. For each vertex *v*∈*V*_*i*_, we build a node gadget *G*_*v*_ as follows. We introduce vertices *v* ^*j*^ for each 0≤*j*≤*k*+1 and a vertex *e*_*v*,*z*_ for each *z*∈*N*_*G*_(*v*). For each *z*∈*N*_*G*_(*v*)∩*V*_*j*_ where *j*<*i* we connect *e*_*v*,*z*_ with *v* ^*j*^ and *v* ^*j*+1^, and for each *z*∈*N*_*G*_(*v*)∩*V*_*j*_ where *j*>*i* we connect *e*_*v*,*z*_ with *v* ^*j*−1^ and *v* ^*j*^. For each *j*∈{1,2,…,*k*}∖{*i*} we denote by *A* ^*j*^(*v*) the vertices connected to both *v* ^*j*^ and *v* ^*j*+1^. Furthermore, we introduce edges *v* ^0^ *v* ^1^, *v* ^1^ *v* ^*k*^, and *v* ^*k*^ *v* ^*k*+1^; this finishes the definition of *G*_*v*_. Notice that each vertex in *V*(*G*_*v*_)∖{*v* ^0^,*v* ^*k*+1^} has even degree, due to the property ensured in the previous paragraph. Moreover, the union of all the sets *A* ^*j*^(*v*) forms an independent set, and every member of this set has degree exactly 2. See Fig. 1 for reference.

Let *G*′ contain the disjoint union of graphs *G*_*v*_ for *v*∈*V*, and let us add the vertex sets *P*={*s*_*i*_,*t*_*i*_∣1≤*i*≤*k*} and *D*={*d*_*x*,*y*_,*d*_*y*,*x*_∣*xy*∈*E*} to *G*′. We connect each *s*_*i*_ with the vertices *v* ^0^ where *v*∈*V*_*i*_, and similarly, each *t*_*i*_ with the vertices *v* ^*k*+1^ where *v*∈*V*_*i*_. In addition, for each edge *xy* in *G* we connect the vertices *e*_*x*,*y*_,*d*_*x*,*y*_,*e*_*y*,*x*_,*d*_*y*,*x*_ in this order via a cycle of length 4. To finish the construction of *G*′, we add the edge *s*_*i*_ *t*_*i*_ in case *s*_*i*_ (and hence *t*_*i*_) would have an even degree otherwise. Notice that the odd degree vertices in *G*′ are exactly the vertices of *P*. Finally, we let the set of forbidden vertices to be *F*={*v* ^*j*^∣*v*∈*V*,1≤*j*≤*k*}∪*D*∪*P*. As the allowed vertices are only the vertices of the form *v* ^0^, *v* ^*k*+1^, or *e*_*x*,*y*_, the claimed property *Δ*_*a*_=4 indeed holds.

First suppose that *X*={*x*_1_,…,*x*_*k*_} is a clique in *G* with each *x*_*i*_∈*V*_*i*_. We prove that *S*={*x* ^0^,*x* ^*k*+1^∣*x*∈*X*}∪{*e*_*x*,*y*_,*e*_*y*,*x*_∣*x*,*y*∈*X*,*x*≠*y*} is a solution for (*G*′,*F*,*k*′). Clearly, |*S*|=*k* ^2^+*k*=*k*′ and *S*∩*F*=∅ hold, so it suffices to show that *G*′∖*S* is an even graph. As *X* contains exactly one vertex from each partition *V*_*i*_, each vertex in *P* has one neighbor in *S*, so the vertices of *P* will become even in *G*′∖*S*. Regarding *D*, only those vertices *d*_*x*,*y*_ and *d*_*y*,*x*_ have a neighbor in *S*, for which *x*,*y*∈*X* holds, and these vertices will lose exactly two neighbors, namely *e*_*x*,*y*_ and *e*_*y*,*x*_, when deleting *S*. A vertex *v* ^*j*^ can only have a neighbor in *S* if *v*∈*X*, and in such a case it is not hard to verify that *v* ^*j*^ will have exactly two neighbors in *S*. Vertices *e*_*x*,*y*_∉*S* do not have neighbors in *S*, so they stay even. This shows that the first direction of the reduction is sound.

For the other direction, suppose that *S* is a solution for (*G*′,*F*,*k*′). For each *i*, the vertex *s*_*i*_ must have at least one neighbor in *S*; suppose that *v* ^0^ is such a vertex. Then, since *v* ^1^ is connected to *v* ^0^ and *N*(*v* ^1^)∖{*v* ^0^}=*A* ^1^(*v*), we know that |*A* ^1^(*v*)∩*S*| is odd. Using that *N*(*v* ^*j*^)∖*A* ^*j*−1^(*v*)=*A* ^*j*^(*v*) for each 2≤*j*≤*k*−1, and that *v* ^1^,…,*v* ^*k*^ are forbidden vertices, we can deduce for each *j*=2,…,*k*−1 that |*A* ^*j*^(*v*)∩*S*| must be odd as well. Hence, *v* ^*k*+1^∈*S* follows. Therefore, if *S* contains a vertex *v* ^0^, then it must contain altogether at least *k*+1 vertices from the node gadget *G*_*v*_. By our bound on the size of *S*, we obtain that *S* contains vertices from exactly *k* node gadgets, and if *S* contains a vertex from *G*_*v*_, then it contains *v* ^0^, *v* ^*k*+1^, and one vertex from each of the sets *A* ^1^(*v*),…,*A* ^*k*−1^(*v*). Let *X* contain those vertices *x* in *G* for which *x* ^0^∈*S*, and let *Y* contain those pairs (*x*,*y*) for which *e*_*x*,*y*_∈*S*. By the previous observations, we know |*Y*|=*k*(*k*−1) and |*X*|=*k*. Suppose that (*x*,*y*)∈*Y*. Note that *x*∈*X* and *y*∈*N*_*G*_(*x*) are immediate from the definition of *G*_*v*_. By looking at the forbidden vertices *d*_*x*,*y*_ and *d*_*y*,*x*_, we get *e*_*y*,*x*_∈*S*, yielding (*y*,*x*)∈*Y*. Therefore, *y*∈*X* as well, and thus each pair in *Y* must contain the end-vertices of an edge connecting two vertices of *X*. By the size of *X* and *Y* we get that *X* must be a clique of size *k*, finishing the proof for the undirected case.

### Directed Case

The reduction for the directed case is very similar to the one used in the undirected case, and hence we only describe the differences. First, we direct the edges of each cycle *e*_*x*,*y*_,*d*_*x*,*y*_,*e*_*y*,*x*_,*d*_*y*,*x*_ in a way that they span a directed cycle. Then for each set *V*_*i*_ of the partition and for each *v*∈*V*_*i*_, we direct every edge *e* incident to a vertex of *G*_*v*_, except for the edge *v* ^1^ *v* ^*k*^, in the direction in which *e* is traversed when going from *s*_*i*_ to *t*_*i*_ through *e* via a shortest path through *G*_*v*_∖{*v* ^1^ *v* ^*k*^}. We remove the edge *v* ^1^ *v* ^*k*^, but for each *v* ^*j*^ with 1≤*j*≤*k*−1 we introduce a certain number of parallel arcs from *v* ^*j*+1^ to *v* ^*j*^ in order to ensure each vertex of *G*_*v*_ to be balanced; this means |*A* ^1^(*v*)|−1 arcs from *v* ^2^ to *v* ^1^, |*A* ^*k*−1^(*v*)|−1 arcs from *v* ^*k*^ to *v* ^*k*−1^, and |*A* ^*j*^(*v*)| arcs from *v* ^*j*+1^ to *v* ^*j*^ for each remaining *j*. As a result, each vertex of *V*(*G*)∖*P* becomes balanced. Finally, we add |*V*_*i*_|−1 arcs from *t*_*i*_ to *s*_*i*_, for each *i*. The set of forbidden vertices *F* and the parameter *k*′ remains unchanged. Also, *Δ*_*a*_=4 remains true.

Clearly, each *s*_*i*_ must lose an outgoing arc and each vertex *t*_*i*_ must lose an incoming arc in a solution. Arguing along similar thoughts as above, one can show that the constructed instance is equivalent with the original one.

In case we want to get rid of parallel arcs, we can simply subdivide each arc contained in a set of parallel arcs with a newly introduced forbidden vertex of degree 2.  □

### Theorem 10

Undirected *and* Directed Eulerian Node Deletion with Forbidden Nodes *remain* W[1]-*hard with parameter* *k*, *even if* *Δ*_*a*_=4.

### Proof

Considering the directed version of the problem, the theorem follows from the proof of Theorem 9.

Consider the construction given in the proof of Theorem 9. Observe that each allowed vertex is only connected to forbidden vertices. Thus, if we ensure that the forbidden vertices remain in one connected component of *G* after deleting an arbitrary set of allowed vertices, then we also ensure that the whole graph remains connected. This can be done by introducing a new forbidden vertex *r*, and connecting *r* to each forbidden vertex by a pair of twin arcs. As each allowed vertex has the same degree as originally, we can conclude that *Δ*_*a*_=4 will still hold in the transformed instance.

For the undirected version we can use the modified version of the reduction above, by connecting each forbidden vertex to *r* using a pair of parallel edges (or two unattached paths of length 2 with middle vertices forbidden) instead of the twin arcs. □

Now we prove that if *every* vertex has bounded degree, then we can solve all of the *α* Node Deletion with Forbidden Nodes problems, even with forbidden nodes, efficiently. The following simple algorithm works:

1. If the parameter *k* is negative, or we aim for an Eulerian induced subgraph and *G* is disconnected, then stop and return NO.
2. If the given graph *G* is already balanced/even/Eulerian, then stop and return the solution collected so far.
3. Otherwise, choose an arbitrary vertex *v* that is not balanced/even, and branch on removing from *G* either *v* or one of its neighbors. In case the deleted vertex *x* was forbidden, stop and return NO in the corresponding branch. Otherwise, put the deleted vertex *x* into the solution, decrease the value of the parameter to *k*−1, and go to Step 1.

The above algorithm can clearly be implemented to run in *O*((*Δ*+1)^*k*^(|*V*(*G*)|+|*E*(*G*)|)) time. Its soundness can be proven easily by induction w.r.t. the size of the solution, using the simple observation that if *v* is a vertex that is not balanced/even, then either *v* or at least one of its neighbors must be in the solution. Thus, we can conclude:

### Theorem 11

*There is an* *O*((*Δ*+1)^*k*^(|*V*(*G*)|+|*E*(*G*)|)) *time algorithm that solves the* *α* Node Deletion with Forbidden Nodes *problem*, *where* *α* *can be* Undirected Even, Undirected Eulerian, Directed Balanced, *or* Directed Eulerian.

## Conclusion

We completed the analysis of the complexity of making a graph Eulerian via edge or vertex deletions. There are two open problems that we would like to emphasize here.

First, do there exist FPT algorithms for the edge-deletions problems running in time *c* ^*k*^ *n* ^*O*(1)^? It seems hard to obtain such algorithms using our techniques, mainly due to the fact that the witness subgraph *W* may contain *Ω*(*k* ^2^) close edges, as was discussed in Sect. 4.2.

Second, Cechlárová and Schlotter in [10] asked for the parameterized complexity of a related problem, where the task is to delete at most *k* arcs from a directed graph to obtain a graph where each strongly connected component is Eulerian. This problem seems to be significantly different than the problems considered in this paper, as for example it includes Directed Feedback Vertex Set [10], and, to the best of our knowledge, the question of its parameterized complexity still remains open.

## Footnotes

1 Strictly speaking, the usual definition of being Eulerian requires only that the graph is connected after removing the isolated vertices. However, we feel that requiring connectivity instead leads to more natural and fundamental problems.
